# Supplementary material for: Return to Work, Fatigue and Cancer Rehabilitation after Curative Radiotherapy and Radiochemotherapy for Pelvic Gynecologic Cancer
Source: Cancers (Basel). 2022 May 8;14(9):2330. doi: 10.3390/cancers14092330 (PMC9099439; doi:10.3390/cancers14092330)
Supplement: Supplementary file 1 [file cancers-14-02330-s001.zip › cancers-1671353-supplementary.pdf]

Table S1. Statistical results for Pearson Chi-squared (X^2) test.

Characteristics	Group1	Group2	Total	*p*-Value
**FIGO stage (1/2 vs. 3/4)** X^2				
acute nausea	44 (43.1%)	58 (56.9%)	102 (24.1%)	<0.001
no acute nausea	204 (63.4%)	118 (36.6%)	322 (75.9%)	
total	1/2: *N* = 248 (58.5%)	3/4: *N* = 176 (41.5%)	424	
acute gastrointestinal toxicity	76 (46.1%)	89 (53.9%)	165 (38.9%)	<0.001 X^2
no acute gastrointestinal toxicity	172 (66.4%)	87 (33.6%)	259 (61.1%)	
total	1/2: *N* = 248 (58.5%)	3/4: *N* = 176 (41.5%)	424	
acute dermatitis/mucositis	42 (54.5%)	35 (45.5%)	77 (18.2%)	<0.001
no acute dermatitis/mucositis	206 (59.4%)	141 (40.6%)	347 (81.8%)	
total	1/2: *N* = 248 (58.5%)	3/4: *N* = 176 (41.5%)	424	
**Chemotherapy (yes vs. no)** X^2				
acute nausea	85 (83.3%)	17 (16.7%)	102 (24.1%)	<0.001
no acute nausea	144 (44.7%)	178 (55.3%)	322 (75.9%)	
total	yes: *N* = 229 (54.0%)	no: *N* = 195 (46.0%)	424	
acute gastrointestinal toxicity	114 (69.1%)	51 (30.9%)	165 (38.9%)	<0.001
no acute gastrointestinal toxicity	115 (44.4%)	144 (55.6%)	259 (61.1%)	
total	yes: *N* = 229 (54.0%)	no: *N* = 195 (46.0%)	424	
acute dermatitis/mucositis	43 (55.8%)	34 (44.2%)	77 (18.2%)	<0.001
no acute dermatitis/mucositis	186 (53.6%)	161 (46.4%)	347 (81.8%)	
total	yes: *N* = 229 (54.0%)	no: *N* = 195 (46.0%)	424	
acute urinary toxicity	67 (60.9%)	43 (39.1%)	110 (25.9%)	0.024
no acute urinary toxicity	162 (51.6%)	152 (48.4%)	314 (74.1%)	
total	yes: *N* = 229 (54.0%)	no: *N* = 195 (46.0%)	424	
acute fatigue	98 (63.6%)	56 (36.4%)	154 (36.3%)	0.015
no acute fatigue	131 (48.5%)	139 (51.5%)	270 (63.7%)	
total	yes: *N* = 229 (54.0%)	no: *N* = 195 (46.0%)	424	
**Charlson Comorbidity Index * (≤5 vs. >5)** X^2				
acute nausea	18 (17.6%)	84 (82.4%)	102 (24.1%)	0.001
no acute nausea	259 (80.4%)	63 (19.6%)	322 (75.9%)	
total	≤5: 349 (82.3%)	>5: 75 (17.7%)	424	
acute fatigue	107 (69.5%)	47 (30.5%)	154 (36.3%)	0.047
no acute fatigue	242 (89.6%)	28 (10.4%)	270 (63.7%)	
total	≤5: 349 (82.3%)	>5: 75 (17.7%)	424	
weight loss during RT	61 (59.2%)	64 (62.1%)	103 (24.3%)	0.005
no weight loss during RT	288 (89.7%)	33 (10.3%)	321 (75.7%)	
total	≤5: 349 (82.3%)	>5: 75 (17.7%)	424	
**Age (<45 vs. ≥45 years)** X^2				
new depression	8 (50.0%)	8 (50.0%)	16 (3.8%)	0.001
no new depression	40 (9.8%)	368 (90.2%)	408 (96.2%)	
total	<45: *N* = 48 (11.3%)	≥45: *N* = 376 (88.7%)	424	
acute urinary toxicity	15 (13.6%)	95 (86.4%)	110 (25.9%)	0.036
no acute urinary toxicity	33 (10.5%)	281 (89.5%)	314 (74.1%)	
total	<45: *N* = 48 (11.3%)	≥45: *N* = 376 (88.7%)	424	
acute fatigue	15 (9.7%)	139 (90.3%)	154 (36.3%)	0.025
no acute fatigue	33 (12.2%)	237 (87.8%)	270 (63.7%)	
total	<45: *N* = 48 (11.3%)	≥45: *N* = 376 (88.7%)	424	
**Smokers (yes vs. no)** X^2				
acute urinary toxicity	21 (19.1%)	89 (80.9%)	110 (25.9%)	0.029

no acute urinary toxicity	38 (12.1%)	276 (87.9%)	314 (74.1%)	
total	yes: N = 59 (13.9%)	no: N = 365 (86.1%)	424	
acute lymph edema	3 (21.4%)	11 (78.6%)	14 (3.3%)	0.011
no acute lymph edema	56 (13.7%)	354 (86.3%)	410 (96.7%)	
total	yes: N = 59 (13.9%)	no: N = 365 (86.1%)	424	
**Presence of children (yes vs. no)** X^2				
acute fatigue	144 (93.5%)	10 (6.5%)	154 (36.3%)	0.006
no acute fatigue	223 (82.6%)	47 (17.4%)	270 (63.7%)	
total	yes: N = 367 (86.5%)	no: N = 57 (13.5%)	424	
**Weight loss before RT (yes vs. no)** X^2				
acute nausea	12 (11.8%)	90 (88.2%)	102 (24.1%)	0.042
no acute nausea	23 (7.1%)	299 (92.9%)	322 (75.9%)	
total	yes: N = 35 (8.3%)	no: N = 389 (91.7%)	424	
acute gastrointestinal toxicity	16 (9.7%)	149 (90.3%)	165 (38.9%)	0.043
no acute gastrointestinal toxicity	19 (7.3%)	240 (92.7%)	259 (61.1%)	
total	yes: N = 35 (8.3%)	no: N = 389 (91.7%)	424	
**Inpatient treatment (yes vs. no)** X^2				
acute fatigue	101 (65.6%)	53 (34.4%)	154 (36.3%)	<0.001
no acute fatigue	36 (13.3%)	234 (86.7%)	270 (63.7%)	
total	yes: N = 137 (32.3%)	no: N = 287 (67.7%)	424	
definitive concept	70 (45.2%)	85 (54.8%)	155 (36.6%)	<0.001
adjuvant concept	67 (24.9%)	202 (75.1%)	269 (63.4%)	
total	yes: N = 137 (32.3%)	no: N = 287 (67.7%)	424	
FIGO stage 1/2	12 (4.8%)	236 (95.2%)	248 (58.5%)	0.006
FIGO stage 3/4	125 (71.0%)	51 (29.0%)	176 (41.5%)	
total	yes: N = 137 (32.3%)	no: N = 287 (67.7%)	424	
extended RT fields	36 (76.6%)	11 (23.4%)	47 (11.1%)	0.012
no extended RT fields	101 (26.8%)	276 (73.2%)	377 (88.9%)	
total	yes: N = 137 (32.3%)	no: N = 287 (67.7%)	424	
Charlson Comorbidity index* ≤ 5	86 (24.6%)	263 (75.4%)	349 (82.3%)	<0.001
Charlson Comorbidity index* > 5	51 (68.0%)	24 (32.0%)	75 (17.7%)	
total	yes: N = 137 (32.3%)	no: N = 287 (67.7%)	424	
**Inpatient rehabilitation (yes. vs. no)** X^2				
acute urinary toxicity	38 (34.5%)	72 (65.5%)	110 (25.9%)	0.031
no acute urinary toxicity	84 (26.8%)	230 (73.2%)	314 (74.1%)	
total	yes: N = 122 (28.8%)	no: N = 302 (71.2%)	424	
High educational level	48 (78.7%)	13 (21.3%)	61 (24.9%)	0.022
Low educational level	12 (6.5%)	172 (93.5%)	184 (75.1%)	
total	yes: N = 60 (28.8%)	no: N = 185 (71.2%)	245	
social in-house counselling	112 (46.5%)	129 (53.5%)	241 (56.8%)	<0.0001
no social in-house counselling	10 (5.5%)	173 (94.5%)	183 (43.2%)	
total	yes: N = 122 (28.8%)	no: N = 302 (71.2%)	424	
fatigue relief	62 (66.0%)	32 (34.0%)	94 (22.2%)	<0.001
no fatigue relief	60 (18.2%)	270 (81.8%)	330 (77.8%)	
total	yes: N = 122 (28.8%)	no: N = 302 (71.2%)	424	
**In-house social service consultation (yes. vs. no)** X^2				
acute nausea	71 (69.6%)	31 (30.4%)	102 (24.1%)	0.011
no acute nausea	170 (52.8%)	152 (47.2%)	322 (75.9%)	
total	yes: N = 241 (56.8%)	no: N = 183 (43.2%)	424	
Charlson Comorbidity index * ≤ 5	193 (55.3%)	156 (44.7%)	349 (82.3%)	0.039
Charlson Comorbidity index * > 5	48 (64.0%)	27 (36.0%)	75 (17.7%)	

total	yes: N = 241 (56.8%)	no: N = 183 (43.2%)	424	
**Return to work (yes vs. no)** X^2				
oncologic progression	4 (11.1%)	32 (88.9%)	36 (18.8%)	<0.001
no oncologic progression	97 (62.2%)	59 (37.8%)	156 (81.3%)	
total	yes: N = 101 (52.9%)	no: N = 91 (47.6%)	192	
FIGO stage 1/2	66 (62.9%)	39 (37.1%)	105 (54.7%)	0.003
FIGO stage 3/4	35 (40.2%)	52 (59.8%)	87 (45.3%)	
total	yes: N = 101 (52.9%)	no: N = 91 (47.6%)	192	
inpatient cancer rehabilitation	79 (64.8%)	43 (35.2%)	122 (63.5%)	<0.001
no inpatient cancer rehabilitation	22 (31.4%)	48 (68.6%)	70 (36.5%)	
total	yes: N = 101 (52.9%)	no: N = 91 (47.6%)	192	
acute fatigue	34 (44.7%)	42 (55.2%)	76 (39.6%)	0.048
no acute fatigue	67 (57.8%)	49 (42.2%)	116 (60.4%)	
total	yes: N = 101 (52.9%)	no: N = 91 (47.6%)	192	
fatigue relief	54 (57.4%)	40 (42.6%)	94 (49.0%)	<0.001
no fatigue relief	47 (48.0)	51 (52.0%)	98 (51.0%)	
total	yes: N = 101 (52.9%)	no: N = 91 (47.6%)	192	
BMI within normal ranges	84 (68.9%)	38 (31.1%)	122 (63.5%)	0.014
Abnormal BMI	17 (24.3%)	53 (75.7%)	70 (36.5%)	
total	yes: N = 101 (52.9%)	no: N = 91 (47.6%)	192	
depression	9 (18.4%)	40 (81.6%)	49 (25.5%)	0.010
no depression	92 (64.3%)	51 (35.7%)	143 (74.5%)	
total	yes: N = 101 (52.9%)	no: N = 91 (47.6%)	192	
definitive concept	42 (40.0%)	63 (60.0%)	105 (54.7%)	
adjuvant concept	59 (67.8%)	28 (32.2%)	87 (45.3%)	0.024
total	yes: N = 101 (52.9%)	no: N = 91 (47.6%)	192	
self-employment	9 (100%)	0 (0%)	9 (6.3%)	0.029
salaried employment	58 (43.6%)	75 (56.3%)	133 (93.7%)	
total	yes: N = 67 (47.2%)	no: N = 75 (52.8%)	142	

SD: standard deviation. RT: radiotherapy, X^2: Pearson Chi-squared (X^2) test. A *p*-value less than 0.05 was being considered statistically significant. * the Charlson comorbidity index [15] was utilized with an adjusted scoring for the category of "solid tumor": points were count-ed only if a second malignancy, other than the treated one, was present.

Table S2. Statistical results for Mann-Whitney U test.

Characteristics	N	Median	Mean	SD	Q1–Q3	Min–Max	*p*-Value
**Number of fractions** ^{M}	102	29	28.4	±4.3	27–30	4–36	<0.001
acute nausea							
no acute nausea	322	27	20.4	±12.1	4–29	1–38	
acute gastrointestinal toxicity	165	29	28.3	±4.5	27–30	4–35	<0.001
no acute gastrointestinal toxicity	259	27	18.5	±12.6	4–29	1–38	
acute dermatitis/mucositis	77	29	27.9	±6.1	27–30	4–36	<0.001
no acute dermatitis/mucositis	347	27	21.0	±11.7	4–29	1–38	
acute urinary toxicity	110	29	26.4	±7.9	27–30	1–36	<0.001
no acute urinary toxicity	314	27	20.8	±11.9	4–29	3–38	
acute fatigue	154	29	26.0	±8.3	27–30	4–36	<0.001
no acute fatigue	270	27	20.1	±12.2	4–29	1–38	
inpatient treatment	137	29	28.8	±3.5	28–30	4–38	<0.001
no inpatient treatment	287	4	15.5	±12.4	4–28	1–35	
inpatient cancer rehabilitation	122	29	24.0	±10.2	25–30	3–35	0.032
no inpatient cancer rehabilitation	302	28	21.4	±11.7	4–29	1–38	
**RT doses** ^{M}							

acute nausea	102	45	46.7	±4.5	45.0–50.4	22.0–54.4	<0.001
no acute nausea	322	45	38.9	±12.8	22.0–50.4	5.5–60.0	
acute gastrointestinal toxicity	165	45	46.7	±4.9	45.0–50.4	22.0–60.0	<0.001
no acute gastrointestinal toxicity	259	45	36.9	±13.3	22.0–50.4	5.5–60.0	
acute dermatitis/mucositis	77	50.4	47.9	±7.3	45.0–50.4	22.0–60.0	<0.001
no acute dermatitis/mucositis	347	45	39.1	±12.1	22.0–50.4	5.5–60.0	
acute urinary toxicity	110	45	44.9	±9.1	45.0–50.4	5.5–56.0	<0.001
no acute urinary toxicity	314	45	39.2	±12.4	22.0–50.4	15.0–60.0	
acute fatigue	154	45	44.7	±9.0	45.0–50.4	22.0–60.0	<0.001
no acute fatigue	270	45	38.5	±12.7	22.0–50.4	5.5–60.0	
inpatient treatment	137	45	46.9	±4.7	45.0–50.4	22.0–60.0	<0.001
no inpatient treatment	287	22	34.3	±13.6	22.0–50.4	5.5–56.0	

[M]: Mann-Whitney U test, max: maximum, min: minimum, N: number of patients, Q1: first quartile, Q3: third quartile, SD: standard deviation. A *p*-value less than 0.05 was being considered statistically significant.
